# High Throughput Proteomic Exploration of Hypothermic Preservation Reveals Active Processes within the Cell Associated with Cold Ischemia Kinetic

**DOI:** 10.3390/ijms22052384

**Published:** 2021-02-27

**Authors:** Ophélie Pasini-Chabot, Julia Vincent, Sylvain Le Pape, Maryne Lepoittevin, Wassim Kaaki, Jean-Baptiste Woillard, Sebastien Giraud, Nicolas Bourmeyster, Thierry Hauet, Raphael Thuillier

**Affiliations:** 1 Inserm U1082, F-86000 Poitiers, France; ophelie-pasini@orange.fr (O.P.-C.); jujuvincent@hotmail.fr (J.V.); sylvain.le.pape@univ-poitiers.fr (S.L.P.); lepoittevin.maryne@gmail.com (M.L.); giraudseb@yahoo.fr (S.G.); thierry.hauet@univ-poitiers.fr (T.H.); 2 CHU Poitiers, Service de Biochimie, Pôle BIOSPHARM, F-86000 Poitiers, France; nicolas.bourmeyster@univ-poitiers.fr; 3 Faculté de Médecine et de Pharmacie, Université de Poitiers, F-86000 Poitiers, France; 4 Eurofins CEREP SA, F-86600 Celle-Lévescault, France; WassimKaaki@eurofins.com; 5 Inserm, U1248 IPPRITT, F-87032 Limoges, France; jean-baptiste.woillard@unilim.fr; 6 Fédération Hospitalo-Universitaire SUPORT, F-86000 Poitiers, France; 7 UFR Sciences Fondamentales et Appliquées, Laboratoire STIM (ERL7268), Université de Poitiers, F-86000 Poitiers, France; 8 IBiSA Plateforme ‘MOPICT’, Institut National de la Recherche Agronomique, Unité Expérimentale, Génétique, Expérimentations et Systèmes Innovants, Domaine Expérimental du Magneraud, F-17700 Surgères, France

**Keywords:** ischemia-reperfusion, organ preservation, cellular mechanism, proteomics, metabolism

## Abstract

The demand for organs to be transplanted increases pressure on procurement centers, to the detriment of organ quality, increasing complications. New preservation protocols are urgently needed, requiring an in-depth understanding of ischemia-reperfusion mechanisms. We performed a proteomic analysis using LC-MS/MS-TOF data analyzed through R software and Cytoscape’s ClueGO application, comparing the proteome of kidney endothelial cells, key cell type, subjected to 3, 6, 12, 19, and 24 h of cold ischemia and 6 h reperfusion. Critical pathways such as energy metabolism, cytoskeleton structure/transport system, and gene transcription/translation were modulated. Important time windows were revealed: a—during the first 3 h, central proteins were upregulated within these pathways; b—the majority of these upregulations were maintained until 12 h cold ischemia time (CIT); c—after that time, the overall decrease in protein expression was observed; d—at reperfusion, proteins expressed in response to cold ischemia were all downregulated. This shows that cold ischemia is not a simple slowing down of metabolism, as deep changes take place within the proteome on major pathways. Time-sensitive expression of key protein reveals possible quality biomarkers as well as potential targets for new strategies to maintain or optimize organ quality.

## 1. Introduction

The rise of transplantation to the status of most adapted treatment for the end-stage renal disease has increased the demand for organs, surpassing the donation rate by several folds. This organ shortage led to the extension of donor criteria and definition of new donor categories: extended criteria donors (ECD) [1], which are donors aged over 60, or donors aged 50–59 with at least two of three additional risk factors including cerebrovascular accident as a cause of death, history of hypertension, and serum creatinine above 1.5 mg/dL prior to transplantation; deceased after circulatory death donors (DCD), in which the organ is subjected to a period of warm ischemia before procurement. Crucial in facing the organ shortage, these new organs are particularly sensitive to ischemia-reperfusion injury (IRI) [2], well defined as having a dramatic impact on short [3,4,5] and long term [6,7] outcome. Enabling these organs to better withstand IRI has thus become a priority in the transplant community, however, efforts to design new protocols for ECD organ preservation are impeded by the lack of mechanistic data on IRI.

Cold ischemia is generally described in simple terms as a stasis condition in which metabolism is slowed to approximately 10–14% of its original level, following the Van ’t Hoff equation and the Arrhenius relation on the efficacy and speed of a chemical reaction, respectively [8,9]. However, a cell is significantly more complex than a test tube and is composed of structures particularly sensitive to cold ischemia [10] such as proteins [11,12] and phospholipid bilayer membranes [13]. Moreover, temperature also influences bonds central to cellular processes, such as the hydrogen bond [14,15] and the hydrophobic effect [16]. In this light, cold ischemia likely induces complex cellular rearrangements.

We and others have actively pursued the definition of IRI mechanisms for some time [17,18]. A recent development in high throughput technologies and system biology [19,20] opens the possibility of an "open-ended" approach in which a lesion can be studied with as broad a viewpoint as possible. Herein, we attempt such an approach to decipher the cellular consequences of cold ischemia using proteomics. An in vitro model is used, with endothelial cells subjected to hypothermia and anoxia in conditions mimicking the stresses of organ preservation: rinsing with cold preservation solution and static storage for up to 24 h. While limited in scope, this model permits a reduction in the number of variables, which is not the case in a multi-cell type organ.

## 2. Results

### 2.1. Data Segregation

**Figure 1:**
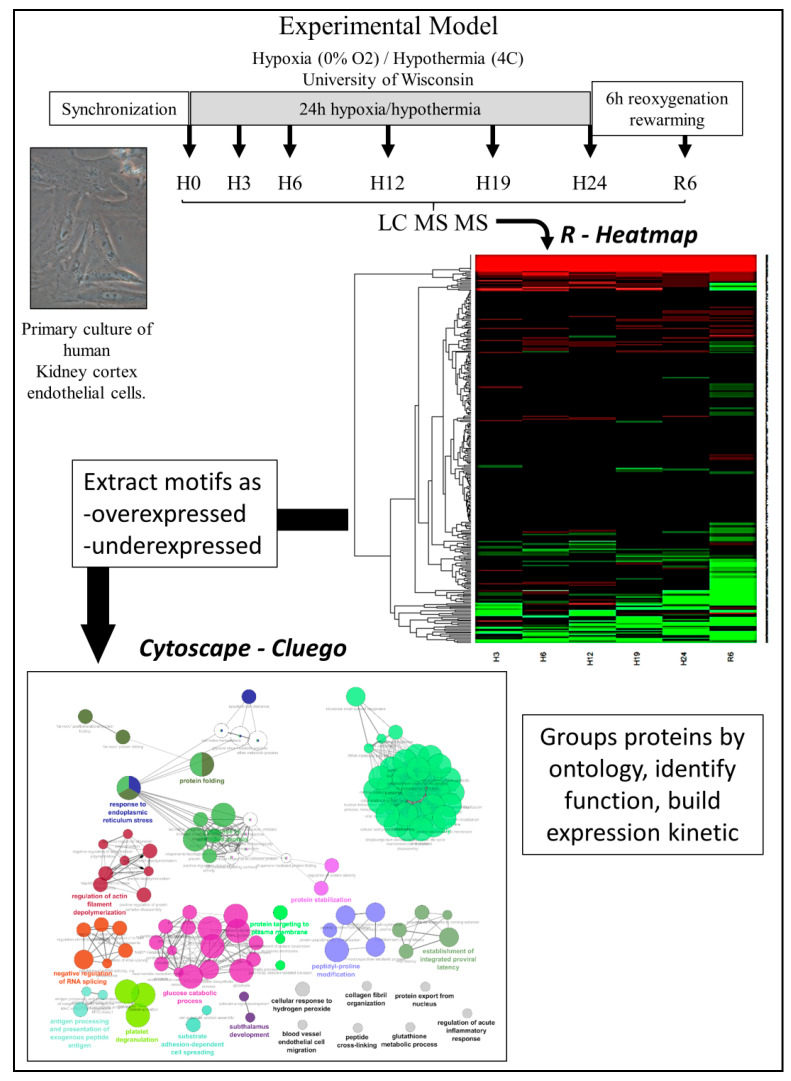
**Experimental model and analysis strategy**. Primary human renal glomerular endothelial cells (HRGEC) were synchronized and were subjected to cold-ischemia like conditions: incubation in a hermetic chamber at 4 °C containing a hypoxic atmosphere: 0% O_2_, 5% CO_2_, and 95% N_2_ for up to 24 h (Hypothermic/Hypoxic period, H), in University of Wisconsin solution, then washed and incubated in regular culture conditions (Reperfusion period, R). At each time point, cell monolayers were collected for analysis. After LC-MS/MS analysis, a heatmap was generated to distinguish protein motifs by their variation, and each group was analyzed using Cytoscape’s ClueGO to identify their ontology and network associations.

The analysis procedure is schematized in Figure 1, 174 human proteins with an MW above 30 kDa were analyzed.

**Figure 2:**
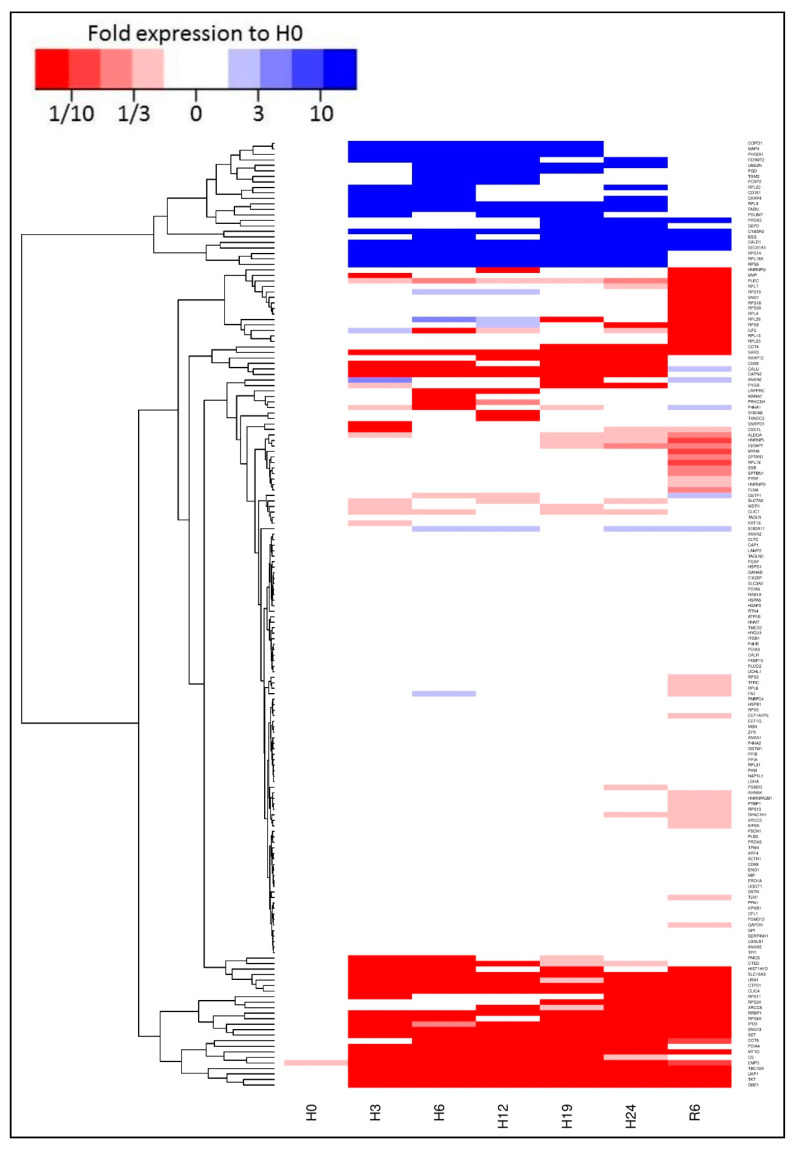
**Proteome heatmap.** A heatmap was generated using the R software, highlighting three groups of protein motifs using as criteria a variation of expression of more than 2 folds between the conditions: proteins unchanged by the protocol (white), protein increasing in expression (Blue), and protein decreasing in expression (Red).

A heatmap was generated (Figure 2) and proteins grouped into 3 categories: proteins unchanged by the protocol (in white), proteins significantly altered by the protocol at any time point (determined by Mann–Whitney U test), either upregulated (in blue) or downregulated (in red).

### 2.2. Unchanged Proteins

**Figure 3:**
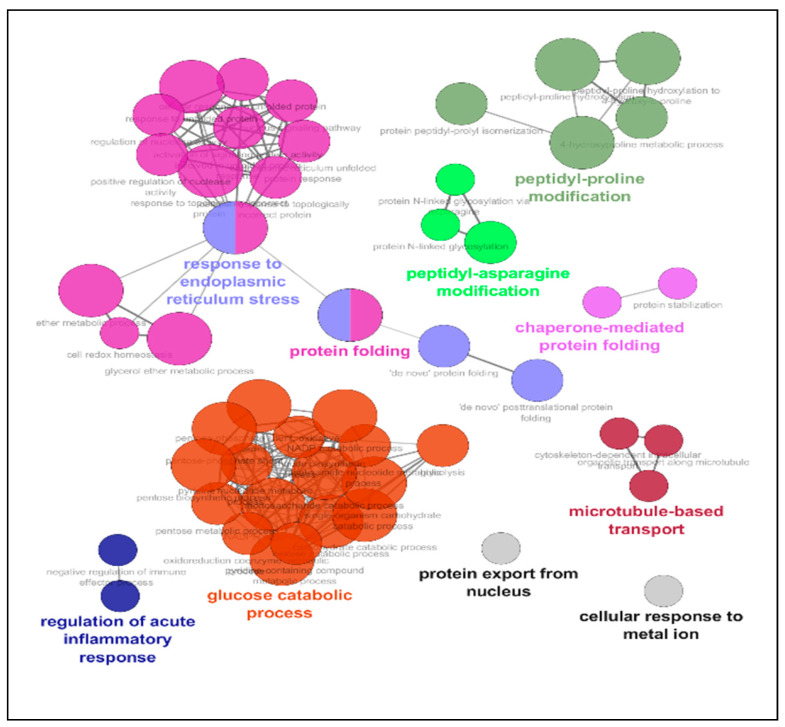
**ClueGO Network Representation of unaltered motifs during the full protocol.** The list of protein motifs unaffected by the protocol were analyzed in Cytoscape’s ClueGO to identify their ontology and network associations. Results are illustrated as a functionally grouped network of terms/pathways. The most significant term of a group is considered to be the leading terms and is highlighted (bolded name). Disc size represents the number of motifs identified in the specified ontology (small: 3–6; medium: 7–9; large: 10 and more).

While the protocol represented drastic alterations of the cell’s environment, a number of motifs remained unaltered (Table 1, Figure 3), belonging to families involved in protein production and folding (Peptidyl-proline modification, Peptidyl-asparagine modification, Protein folding, Protein stabilization) and in protein transport (Microtubule-based transport, Protein export from the nucleus). Another represented pathway was glycolysis, the key to energy metabolism.

### 2.3. Proteins Affected by Anoxic Hypothermia

#### 2.3.1. H0–H3

**Figure 4:**
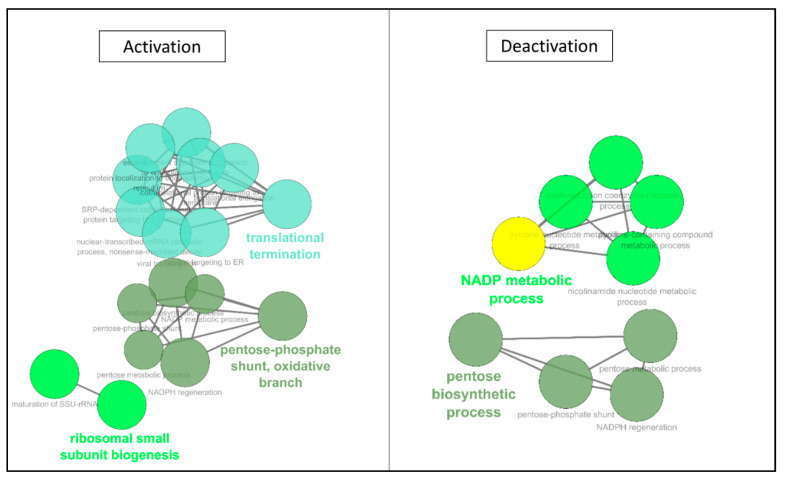
**ClueGO Network Representation of Affected motifs between 0 and 3 h of hypothermic hypoxia.** The list of protein motifs unaffected by the protocol were analyzed in Cytoscape’s ClueGO to identify their ontology and network associations. Results are illustrated as a functionally grouped network of terms/pathways. The most significant term of a group is considered to be the leading terms and is highlighted (bolded name). Disc size represents the number of motifs identified in the specified ontology (small: 3–6; medium: 7–9; large: 10 and more).

In the first 3 h of the protocol, ontology analysis (Table 2), we observed de novo expression of several proteins, belonging to cytoskeletal structure and cellular adhesion. The majority of changes were detected in the transcription/translation machinery, with ribosomal proteins and RNA and peptide transports. The ionic balance was also concerned by new protein productions. In terms of downregulated motifs, both the energy metabolism and nucleotide biosynthesis were affected, as well as cell surface adhesion and RedOx regulation. Network analysis (Figure 4) confirmed the increased expression of protein motifs involved in RNA maturation (translation and ribosome biogenesis), as well as the impact on the energy metabolism, with the reconfiguration of the pentose biosynthetic process, and decrease of the NADPH metabolic process.

#### 2.3.2. H3–H6

**Figure 5:**
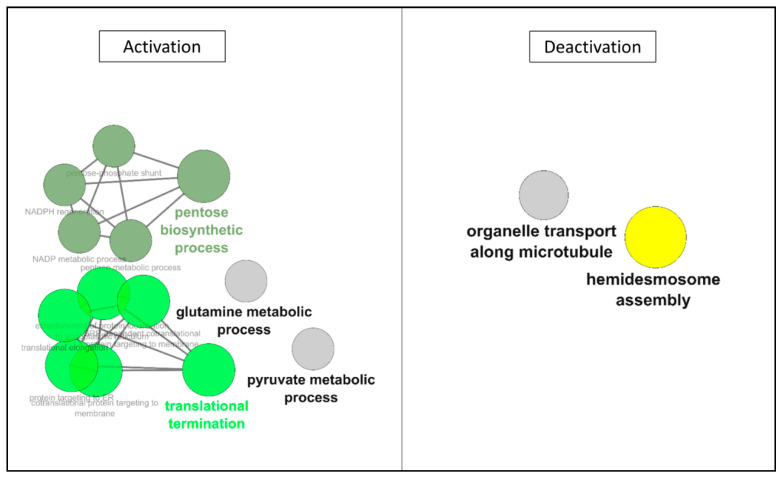
**ClueGO Network Representation of Affected motifs between 3 and 6 h of hypothermic hypoxia.** The list of protein motifs unaffected by the protocol were analyzed in Cytoscape’s ClueGO to identify their ontology and network associations. Results are illustrated as a functionally grouped network of terms/pathways. The most significant term of a group is considered to be the leading terms and is highlighted (bolded name). Disc size represents the number of motifs identified in the specified ontology (small: 3–6; medium: 7–9; large: 10 and more).

Prolonging hypothermic anoxia, ontology analysis (Table 3) showed that ionic balance regulation was also represented in the de novo expression. Whereas in the "interaction with the environment" family, the CD151 antigen produced during H0–H3 was downregulated. Interestingly, the ontologic families "Transcription/Translation Regulation" and "Cytoskeleton" were represented in both downregulated and upregulated motifs categories. Network analysis (Figure 5) demonstrated that reprogramming of RNA maturation and energy metabolism was ongoing, as well as a negative impact on cytoskeletal features.

#### 2.3.3. H6–H12

Reaching the half-time point (Table 4), ontology analysis showed three families still represented in the de novo motif synthesis section: cytoskeleton, the interaction between the cell and its environment which is closely linked to the cytoskeleton, and Transcription/Translation Regulation. The downregulated motifs remain limited, highlighting intracellular priority reshuffling with families such as transport and cell adhesion. The number of motifs were insufficient to conduct network analysis with ClueGO.

#### 2.3.4. H12–H19

**Figure 6:**
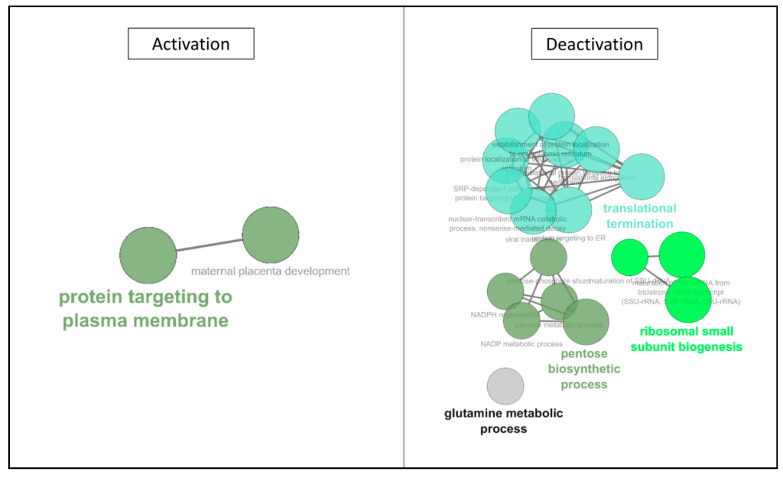
**ClueGO Network Representation of Affected motifs between 12 and 19 h of hypothermic hypoxia.** The list of protein motifs unaffected by the protocol were analyzed in Cytoscape’s ClueGO to identify their ontology and network associations. Results are illustrated as a functionally grouped network of terms/pathways. The most significant term of a group is considered to be the leading terms and is highlighted (bolded name). Disc size represents the number of motifs identified in the specified ontology (small: 3–6; medium: 7–9; large: 10 and more).

In this time window, the exploration of ontologies (Table 5) showed seemed to be a shift in the cell’s behavior. While there remained a few upregulations, with similar families as previous time windows such as intracellular transport, Transcription/Translation Regulation, and interaction with the environment, here energy metabolism was also concerned. The main alterations are however observed in the downregulated section; with a large number of motifs belonging to families which were upregulated in the first half of the procedure: cytoskeleton, interaction with the environment, intracellular transport, and importantly Transcription/Translation Regulation. Ionic imbalance, Energy metabolism, and RedOx regulation were also implicated. Network analysis (Figure 6) confirmed that activations appeared reduced, limited to cytoskeleton-related networks, whereas deactivations were more widespread, with networks previously upregulated during the first 12 h.

#### 2.3.5. H19–H24

Reaching the end of the preservation time (Table 6), we observed the same phenomenon, with only a few upregulated motifs and a majority of downregulated proteins with the major families: Cytoskeleton and Transcription/Translation Regulation as well as energy metabolism and the proteasome. The number of motifs were insufficient to draw a network with ClueGO.

### 2.4. Protein Alteration at Rewarming/Reoxygenation

**Figure 7:**
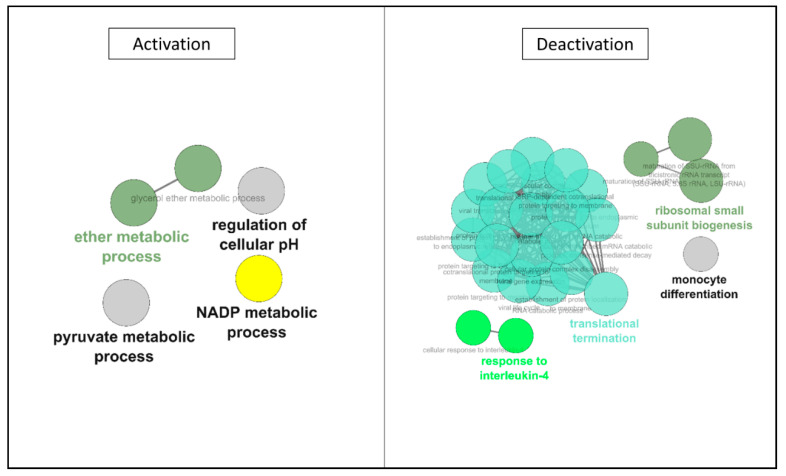
**ClueGO Network Representation of Affected motifs after 6 h reoxygenation/rewarming.** The list of protein motifs unaffected by the protocol were analyzed in Cytoscape’s ClueGO to identify their ontology and network associations. Results are illustrated as a functionally grouped network of terms/pathways. The most significant term of a group is considered to be the leading terms and is highlighted (bolded name). Disc size represents the number of motifs identified in the specified ontology (small: 3–6; medium: 7–9; large: 10 and more).

After 6 h of being cultured back in normal conditions (Table 7), we observed an important downregulation of a large number of motifs while no new proteins are detected. The majority of motifs being downregulated belong to cytoskeleton and Transcription/Translation Regulation families, and other represented families are energy metabolism, interaction with the environment, and ionic balance regulation. Network analysis (Figure 7) confirmed this, notably showing activation of previously deactivated pathways (such as NADP and pyruvate processes), and particularly demonstrating deactivation of pathways specifically induced by ischemia, specifically RNA translation reprogramming.

### 2.5. Kinetic of the Protein Expression Alteration during the Procedure

**Figure 8:**
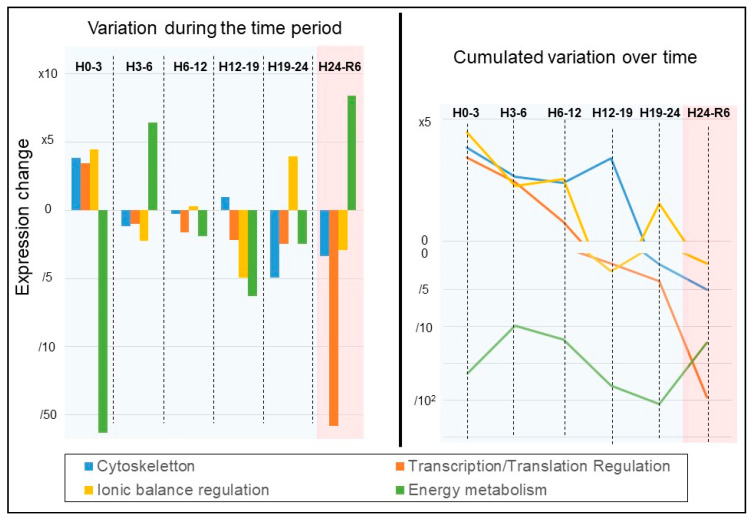
**Kinetics of protein expression during the procedure, by ontology.** We averaged the expression change for the proteins belonging to the most represented ontologies: Cytoskeleton, Transcription/Translation Regulation, Ionic Balance Regulation, Energy metabolism. Left Panel: average change reported for each time period independently. Right Panel: cumulated change in expression over time. (N = 3, *n* = 3).

To obtain a global view of the alterations over time, we represented the average change in expression for each ontology in two different fashion (Figure 8): either the change observed in each time period independently (left panel) or the cumulated change (right panel). We performed this analysis for each of the four most affected ontologies: Cytoskeleton, Transcription/Translation Regulation, Ionic Balance Regulation, Energy metabolism.

Two patterns were observable:

1—In response to the drastic changes in conditions, important de novo expression was taking place within the cell (H0–H3). This increase was not found afterward, however, the level was maintained for a certain duration. After a certain time period, protein expression collapsed. Interestingly, all three ontologies showing this pattern did not show the same kinetic. The first to start decreasing was the Transcription/Translation Regulation: as soon as 3 h were passed, the expression level tends to decrease, a trend which worsened over time. The ionic balance regulation maintained its higher expression levels until 12 h, then collapsed. The Cytoskeleton was the last to collapse, starting at H19. This pattern was also characterized by a further lowering of protein expression levels at reoxygenation.

2—In response to hypothermia and anoxia, the expression level of proteins belonging to the energy metabolism ontology collapsed; however, it was soon followed by de novo expression of protein motifs, which remained higher until 12 h, upon which expression collapsed again. At reoxygenation, a major increase in protein expression was recorded.

### 2.6. Remarkable Protein Motifs Affected by Ischemia-Reperfusion

Within the modulated motifs, across all the time point, several are prominent:

#### 2.6.1. Cytoskeleton Ontology

Plectin: an intermediate filament-associated protein, acting as a cytoskeletal crosslinker and signaling scaffold, was downregulated between 6 and 12 h.

S100A6: a member of a superfamily of EF-hand Ca^2+^-binding proteins, was upregulated between 3 and 6 h, remained stable until 19 h of preservation conditions, then further increased, one of the very few motifs doing so at that time.

#### 2.6.2. Transcription/Translation Ontology

A detailed observation of the impacted motifs shows that the majority coding for ribosomal proteins, accompanied by transcription factors, RNA and protein adaptor/elongation proteins, and even histones. Among the later:

- Interleukin-2 enhancer-binding factor 2 (ILF2), is one of the proteins being overexpressed at the start, with the maintenance of this expression level until 12 h, upon which it is further overexpressed whereas the majority of motifs in this ontologic family are depressed. Only after 19 h do we observe the downregulation of ILF2.
- Eukaryotic translation elongation factor 1 gamma (EEF1G) is a component of the translation apparatus, the eEF1 complex. We observe that its expression is constant at first then overexpressed between 6 and 12 h, before being downregulated constantly until the end of the procedure.
- H2A histone family member X (H2AFX, also known as H2AX), an important member of the DNA damage detection and repair apparatus, is one of the only two motifs detected to be overexpressed between 19 and 24 h of preservation.

#### 2.6.3. Energy Metabolism Ontology

Of the modulated motifs, we note:

- Transketolase (TKT), a key enzyme of the pentose phosphate pathway, was upregulated between 3 and 6 h and downregulated after 12 h.
- TBC1D4, a Rab GTPase-activating protein, was upregulated between 3 and 6 h and downregulated after 12 h.
- Prosaposin (PSAP), a precursor to saponins, was overexpressed between 12 and 19 h.

#### 2.6.4. Ionic Balance Ontology

One of the most affected ontology family with several markers of interest:

- Neuroblast differentiation-associated protein AHNAK, also known as desmoyokin, showed a maintained expression until 12 h, after which it is recorded as decreased both between 12 and 19 h as well as between 19 and 24 h.
- Annexin A6 (ANXA6), a member of the annexins family of Ca^2+^ dependent phospholipid-binding proteins, was upregulated as soon as the cell was subjected to organ preservation conditions, likely related to pH alterations, and remained elevated until 12 h of preservation.

## 3. Discussion

We describe herein an attempt at exploring the proteomic implications of cold ischemia at the cellular level. To our knowledge, this is the first study of its kind. We used a simple model of in vitro cold ischemia, mimicking the conditions withstood by an organ during transport, namely: hypothermia (4 °C), anoxia and preservation solution (UW). We used a primary culture of endothelial cells, as these represent the first cell impacted by IR. While this limits the scope of our study in terms of complexity, the simplicity of the model is a strength in terms of reproducibility. Moreover, in our hands, this model has been used to test compounds against IR before full preclinical testing on large animals, and the level of superposition between in vitro and in vivo results was significant [7,21,22].

We first processed the data using a heatmap. There was a large number of motifs for which expression was not altered. These belonged to ontologies related to protein production, folding, and transport as well as energy metabolism. This suggests that even though extreme conditions of temperature and oxygen pressure, the cell retains a general array of proteins in pathways that are central to its survival. Thus, alteration of its physiology will likely take place through either expression modulation in key elements of the chain, or post-translational regulation (phosphorylation, cleavage, etc.).

We next focused on proteins specifically modulated by our conditions. To this end, we used Cytoscape’s ClueGo application [23]. This showed that while several families were common between the time points, there were individual differences. This implies that cold ischemia not only changes the proteome by turning off certain pathways and inducing new ones but also alters the protein present within the same regulatory pathway, hence reprogramming it. We recorded four ontologies that were majorly impacted: Cytoskeleton, Transcription/Translation Regulation, Ionic Balance Regulation, and Energy metabolism. The first three demonstrated a similar pattern, albeit with different timing: heightened protein expression in the first hours of the procedure, followed by maintenance of this expression then collapse after a certain time point. This implies that the cell is capable of de novo expression of proteins, even while subjected to hypothermia and anoxia, but that each ontology has either differing energy needs or a differing priority in terms of cell health. Observing the general variations in protein expression, it appears that 12 h represents a key limit in our model, after which we observe major downregulations. Interestingly, in our hands, 12 h is the preservation time after which reperfusion induces cell death, with an intensity proportional to the time of preservation [24]. This is concordant with the now well-defined correlation between cold ischemia time, ischemia-reperfusion, and long-term adverse outcomes [25].

Several protein motifs were singled out during the analysis.

Regarding the cytoskeleton, the impact on Plectin, which affects mechanical as well as dynamic properties of the cytoskeleton [26], is coherent considering the observed impact of organ preservation conditions on cell polarity and cytoskeleton [27]. S100A6 was one of the few proteins that constantly increased, likely due to its role in regulating a wide array of cellular and molecular functions, including cell proliferation, differentiation, and survival as well as Ca^2+^ dynamics [28]. Indeed, S100A6 has been functionally linked to changes in cellular motility and cytoskeletal reorganization [29], and S100A6 gene therapy improved survival, infarct size, and viability in acute heart I/R injury models [30]. This target could thus be an interesting avenue of research to improve graft quality.

Regarding motifs belonging to the Transcription/Translation ontology, the fact that ribosome subunits were impacted is an intriguing aspect of our results, indeed ribosomes composition is seldom considered. However, not all mRNA are considered equal by ribosomes, and some RNA molecules are only translated under altered conditions, as in the case of internal ribosome entry sites [31] or upstream open reading frames [32] mRNAs. Hence, it is likely that ribosome subunit composition alters the affinity for different mRNAs and favors the translation of specific ones. Moreover, ribosome composition may also affect ATP consumption [33], an area yet little explored, which could unveil interesting mechanisms of adaptation to low ATP conditions such as found during ischemia. Passive selection of proteins through decreased ATP availability, possibly through ribosome re-configuration, could represent an interesting area of future research in cellular adaptation and survival.

Other interesting motifs in this ontology were: ILF2, involved in the regulatory subunit of NF90/NF110 complexes, themselves implicated in mitotic control [34], and RNA metabolism aspects such as transcription, transport, stability, and translation [35]. ILF2 interacts with RNA-binding proteins (RBPs) involved in DNA repair, genome stability maintenance, and DNA damage response [36]. Its early overexpression and long maintenance suggest a central involvement of this protein in preservation injury. EEF1G functions in the transport of aminoacyl tRNAs to the ribosome for protein synthesis. Interestingly, EEF1G also plays a role as a transcription factor, through interaction with the RNA polymerase II and shuttling or nursing mRNA [37]. Moreover, the N-terminal region of the eEF1G protein contains a glutathione transferase domain [38], which could play an active role in glutathione usage as a defense mechanism. Maintenance of its expression at the beginning of the procedure highlights its role in RNA translation, and the peak of expression before 12 h underlines the importance of this time point in regard to survival. -H2A is particularly involved in the identification of DNA breaks [39] and in hypoxic situations, it promotes endothelial cell proliferation and is necessary for proper neovascularization [40]. This suggests that DNA protection remains a priority for the cell to the very end and that H2AFX could be an interesting target for organ evaluation and therapy.

Among Energy Metabolism ontology motifs we identified: TKT, which activity is increased in cells with heightened energy needs, such as cancer cells [41], while knockdown of TKT suppresses NAPDH production, increases ROS production, and inhibits the cell cycle [42]. Interestingly, the TKT promoter contains multiple stress-inducible control sequences [43]. Hence, TKT alteration could represent the answer of the cell to the extreme change in conditions and its impact on energy metabolism motifs recorded between 0 and 3 h and could be a precious pathway to investigate to improve preservation quality. TBC1D4, which controls intracellular trafficking of protein-bearing membrane vesicles [44], among which the glucose transporter GLUT4 [45]. It also interacts with the Na^+^-K^+^-ATPase α-subunit, inducing intracellular retention of the Na^+^-K^+^-ATPase, targeting ionic balance [46,47]. Its modulation suggests that the cell’s answer to stress not only includes reprogramming of the energy metabolism but also favors glucose intake and controlled ionic balance. PSAP, located in lysosomes and enhancing lysosomal hydrolytic activities, notably the degradation of glycosphingolipids into short oligosaccharides; while it also functions extracellularly as an activator of the Akt and ERK pathways [48] and can protect the cell against oxidative stress-induced cell death [49]. PSAP was one of the few proteins to be upregulated between 12 and 19 h, likely representing an effort from the cell to obtain energy from any available resource, as well as promote survival through paracrine mechanisms.

Finally, within the ionic balance regulation ontology, we identified: -AHNAK, involved in the maintenance of the structural and functional organization of the subsarcolemmal cytoarchitecture [50], located within specific vesicles called “enlargosomes,” and is redistributed to the external surface of the plasma membrane in response to large increases in Ca^2+^, participating in cell membrane differentiation and repair [51]. Regulation of AHNAK after 12 h suggests that this protein could be directly involved in the ability of the cell to withstand the harsh conditions of preservation and a key factor in the “12 h limit” after which organ quality collapses. -ANXA6, participates in the transduction of intracellular Ca^2+^ which, along with acidic pH and cholesterol, regulates the membrane targeting for this annexin [52], where it functions as membrane organizer and channel modulator [53]. We observe upregulated as soon as the cell was subjected to organ preservation conditions, likely related to pH alterations, and it remained unchanged 12 h of preservation, hinting towards a role as a factor in the “12 h limit” discussed above.

Our results highlight the fact that cold ischemia cannot be assimilated to a simple slowing down of the cell metabolism. Indeed, in-depth changes take place within the proteome, with the reprogramming of major areas such as the cytoskeleton, energy metabolism, and ionic balance. Furthermore, we demonstrate that this injury impacted the protein-producing machinery at every step of the process, from DNA folding to RNA maturation and ribosome composition and regulation. Our results show an interesting degree of similarity with a previous study from our group in which we performed transcriptomic analysis of the kidney after warm ischemia (more than 100 altered transcripts) and after subsequent cold storage (more than 400 altered transcripts) [54]. One of the findings was that several RNA coding for proteins involved in protein folding were upregulated (for instance Heat shock protein 70 kDa and thioredoxin-related transmembrane protein 4) and the proteasome (for instance Proteasomal ATPase-associated factor 1) [54], highlighting that ischemia did indeed induce the protein production and quality check machinery. Several parallels can be drawn between the two studies. Cytoskeleton: several transcripts of the Rho family GTPases were altered. Transcription/Translation: elements of the chain, for instance, several histones (also observed herein): Histone cell cycle regulator (HIRA) and Histones 2A and 4, as well as DNA damage response machinery (Early growth response protein 1, Kruppel-like factor-4 and Polo-like kinase 3) and RNA metabolism (RNA methylation PAF1 complex). Energy metabolism: here also, several transcripts were regulated, such as elements of the MAPK pathways.

While the models were different (in vitro culture herein and porcine kidneys in the transcriptome study), the interesting degree of concordance between the result sets suggests some level of conservation in the pathways induced by ischemia-reperfusion. Such an intermediate level of concordance between the studies may be due to one of the limits of our study, namely the fact that we focused on a single cell type, cultured in 2D. This scale limitation permitted a high level of control over sample time and sample concordance, but significance can be lost. Specific programs, both in terms of endothelial cell origin and larger organ experiments, will need to be conducted to consolidate the concepts uncovered herein. Our work opens the possibility to further investigation in models proposing a higher level of sophistication [55]. Such a model would permit for instance to mimic perfusion, such as with microfluidic chambers, as the use of machine perfusion is increasing, particularly for marginal donors, and provide answers to the rising interest in designing novel therapeutics compatible with this mode of preservation [56]. Further complexification could include multiple cell types constructs, such as co-culture of primary cells or the higher sophistication of organoids [57], which could also be included in microfluidic chambers, paving the way for the reproduction of the level of complexity found in animals, using circulating cells to reproduce the first stage of immune activation.

Another limit is that we did not perform reperfusion at every time point studied, hence could not evaluate cell recovery for each level of ischemia. Indeed, due to the amount of data generated and quantity of work required to perform these analyses, we focused on the kinetics of ischemia, with a special interest in exploring the relationship between time and impact, as recent work has hinted towards a "threshold" after which organ quality rapidly degrades. We indeed performed reperfusion experiments in which ischemia time was gradually increased, with the same cells, and demonstrated that a shift was occurring between 6 and 12 h [24]. Endeavoring to explore this shift herein, our experiments brought further clarification to the "12-h limit". However, further definition of this time limit will have to be explored with models of both ischemia and reperfusion, to determine the consequences of the observed proteomic changes on cell phenotype post-reperfusion to contribute to the improvement of organ monitoring strategies.

While it has been described that ischemia has an impact on the mitochondria [58,59] and the cytoskeleton [60], with the effects on ionic imbalance, have been explored elsewhere [17], our work provides the opportunity to integrate these pathways in a kinetic fashion along the length of 24 h ischemia, a duration compatible with standard clinical times. Moreover, the well-described association between these pathways and cell death pathways (apoptosis, necrosis) as well as the activation of the immune system [61] provides increased quality to the description of ischemia-reperfusion injury, a key issue in transplantation as well as other pathologies. Among these pathways, recent work highlights the interplay between NADP and mTOR in survival and protection against ischemia-reperfusion [62,63] as well as the benefits of NADPH supplementation [64]; this is concordant with our results regarding the NADP pathways and the role of TKT. Moreover, we demonstrate that RhoGTPases are impacted, a pathway recently highlighted to play a critical role in kidney disease, notably podocytopathy [65]. Hence, by describing such early pathways affected by ischemia, our results uncover therapeutic possibilities upstream from final pathways such as cell death and immune activation.

After 6 h of reperfusion, no new proteins were detected, however, we observed an important downregulation of a large number of motifs. Interestingly, the motifs being downregulated had not been detected during hypothermic anoxia. Thus, we may not necessarily observe a retraction of the defense mechanisms put in place during stress, but another reshuffling of priorities: cells having survived the conditions of organ preservation must now repair and regenerate to regain the pre-preservation state.

The parallels between transcriptomics and proteomics results confirm that the cell and the organ are not inactive in response to organ preservation and that pathways exist to increase resiliency and possibly quality, which directly impacts the outcome. Hence, specific programs will need to be designed towards a better understanding of the areas uncovered herein to improve our knowledge of the physiopathological mechanism of IRI and be able to devise new protocols to improve organ resistance and thus the success of transplantation. Finally, data strengthen the fact that ischemia is a key step during the transplantation process with important transcriptional and proteome modifications inducing a full reprogramming of major survival/response pathways.

## 4. Materials and Methods

### 4.1. Materials and Sample Preparation

Primary human renal cortex endothelial cells (HRGEC, CliniSciences, Nanterre, France) were used as previously published [24]. At 80% confluence and after synchronization (**H0**), cells were subjected to cold ischemia like conditions: incubation in a hermetic chamber at 4 °C containing an anoxic atmosphere: 0% O_2_, 5% CO_2_, and 95% N_2_ (Bactal 2 gaz, Air Liquide, Paris, France) during 3, 6, 12, 19 and 24 h (**H3, H6, H12, H19, H24**), in University of Wisconsin solution (UW, Bridge to Life, London, UK). At the end of the 24 h conservation period, the cells were washed and incubated in M200/2% FBS for 6 h (**R6**). At each time point, the cells were rapidly washed, scrapped, and frozen until analysis. 3 independent experiments were performed.

Cell pellets were lysed in a trypsin buffer (ThermoFisher, Illkirch, France) and centrifuged to eliminate cellular debris. A 30 kDa cut-off spin column (MerkMillipore, Fontenay sous Bois, France) was used to alleviate noise.

### 4.2. LC-MS Parameters

5 µL of protein extract was injected into a C18 column, 300 Ǻ, 2.1 mm × 150 mm (Sigma, Saint-Quentin Fallavier, France) maintained at 60 °C. LC-MS was performed with an Aquity UPLC system connected on-line with a Waters Xevo G2-XS-TOF mass spectrometer (Waters, Guyancourt, France). The following gradient was used at a flow rate of 0.2 mL/min: isocratic hold at 5% B for 3 min followed by three steps of linear increases to 25% B at 4 min, 55% B at 34 min, 80% B at 36 min, followed by an isocratic hold at 80% B for 9 min. The column was finally equilibrated with 5% B for 10 min prior to the next run. Solvent A was 99.9% H_2_O/0.1% formic acid and solvent B was 90% ACN/9.9% H_2_O/0.1% formic acid.

MS source parameters were: ESI capillary voltage +4.3 kV, desolvation temperature 350 °C, cone gas flow rate 10 L/min, nebulizer pressure 25 psig, and fragmentor voltage 250 V. Data were acquired at 1 spectrum/sec, with acquisition window 100 to 3000 m/z.

### 4.3. Data Analysis

Raw data were processed using the PLGS software (Protein Lynx Global Server, Waters), interrogating the Uniprot database. As the MS software provided us with a list of uniprot identifiers attached to a signal intensity per sample, we first tackled the issue of normalization. All signals were expressed as a ratio to the mean signal at H0, to show variation to baseline. Then, selecting the motifs identified in homo sapiens, we adapted a strategy similar to that of the gNorm algorithm in QPCR data management [66]: a shortlist of the most stable proteins was compiled with proteins a) detected in all samples, and b) with the smallest SD (expressed as % of average signal to average H0). This list counted: VIM, CLTC, LGALS1, PDIA6, PPIA, PFN1, MIF, CD99, P4HA2, SCARB2. Then, signal (ratio to mean H0) from these 10 proteins was log-transformed, normalized by each of the 9 others, the SD calculated for each normalization and the average of these SD was termed "M". The 9 proteins with the lowest M were then selected, and the procedure was repeated until the most stable were identified: VIM et SCARB2. The evolution of M is shown in Supplementary Figure S1. Normalization of each signal was performed with the following formula:

$$\frac{\left(signal\;intensity\;H/\mathrm{moy}\;\mathrm{signal}\;\mathrm{intensity}\;H0\right)}{\sqrt[{f}]{\prod_{0}^{f}\left(ref\;signal\;intensity\;Hx-\mathrm{moy}\;\mathrm{signal}\;\mathrm{intensity}\;H0\right)}}$$

The signal was then log10 transformed for a better estimation of fold change in either direction.

### 4.4. Statistics

The R software was used for statistical analysis and heatmap generation. The significance of variations from one-time point to the next was evaluated using the non-parametric Mann–Whitney U test. Each time point was repeated 3 times (N = 3, *n* = 3).

## Figures and Tables

**Table 1:** Proteins which expression was maintained throughout the course of hypoxia/hypothermia and reoxygenation.

Protein ID	Protein Name	Protein Function
*Peptidyl-proline modification*		
FKBP10	peptidyl-prolyl cis-trans isomerase FKBP10	Accelerates the folding of proteins during synthesis.
P4HA1	Prolyl 4-hydroxylase subunit alpha-1	Quaternary maturation: Catalyzes the post-translational formation of 4-hydroxyproline in -Xaa-Pro-Gly- sequences in collagens and other proteins.
P4HA2	prolyl 4-hydroxylase subunit alpha-2	Quaternary maturation: Catalyzes the post-translational formation of 4-hydroxyproline in -Xaa-Pro-Gly- sequences in collagens and other proteins.
P4HB	protein disulfide-isomerase	Catalyzes the formation, breakage, and rearrangement of disulfide bonds.
PPIA	peptidyl-prolyl cis-trans isomerase A	Accelerates the folding of proteins during synthesis.
PPIB	Peptidyl-prolyl cis-trans isomerase B	Accelerates the folding of proteins during synthesis.
*Peptidyl-asparagine modification*		
CALR	Calreticulin	Calcium-binding chaperone that promotes folding, oligomeric assembly, and quality control in the endoplasmic reticulum (ER).
GANAB	neutral alpha-glucosidase AB	Catalyzes the maturation of oligosaccharide precursors on proteins.
PDIA3	protein disulfide-isomerase A3	Catalyzes the rearrangement of -S-S- bonds in proteins.
UAP1	UDP-N-acetylhexosamine pyrophosphorylase	Converts UTP and GlcNAc-1-P into UDP-GlcNAc, and UTP and GalNAc-1-P into UDP-GalNAc.
UGGT1	UDP-glucose: glycoprotein glucosyltransferase 1	Provides quality control for glycoprotein folding in the endoplasmic reticulum.
*Protein folding*		
CALR	Calreticulin	Calcium-binding chaperone that promotes folding, oligomeric assembly, and quality control in the endoplasmic reticulum (ER).
CCT4	T-complex protein 1 subunit delta	Molecular chaperone; assists the folding of proteins upon ATP hydrolysis.
CCT8	T-complex protein 1 subunit theta	Molecular chaperone; assists the folding of proteins upon ATP hydrolysis.
ERO1A	ERO1-like protein alpha	Oxidoreductase is involved in disulfide bond formation in the endoplasmic reticulum.
FKBP10	peptidyl-prolyl cis-trans isomerase FKBP10	Accelerates the folding of proteins during synthesis.
GANAB	neutral alpha-glucosidase AB	Catalyzes the maturation of oligosaccharide precursors on proteins.
HSPA5	78 kDa glucose-regulated protein	Involved in the correct folding of proteins and degradation of misfolded proteins
HSPD1	60 kDa heat shock protein, mitochondrial	Chaperonin is implicated in mitochondrial protein import and macromolecular assembly.
P4HB	protein disulfide-isomerase	Catalyzes the formation, breakage, and rearrangement of disulfide bonds.
PDIA3	protein disulfide-isomerase A3	Catalyzes the rearrangement of -S-S- bonds in proteins.
PDIA6	protein disulfide-isomerase A6	Chaperone that inhibits aggregation of misfolded proteins.
PPIA	peptidyl-prolyl cis-trans isomerase A	Accelerates the folding of proteins during synthesis.
PPIB	Peptidyl-prolyl cis-trans isomerase B	Accelerates the folding of proteins during synthesis.
TXNDC5	Thioredoxin domain-containing protein 5	Possesses thioredoxin activity
UGGT1	UDP-glucose: glycoprotein glucosyltransferase 1	Provides quality control for glycoprotein folding in the endoplasmic reticulum.
TLN1	Talin-1	Probably involved in connections of major cytoskeletal structures to the plasma membrane.
HYOU1	Hypoxia up-regulated protein 1	Plays a role as a molecular chaperone and participates in protein folding.
SERPINH1	serpin H1	Involved as a chaperone in the biosynthetic pathway of collagen.
*Cellular response to metal ion*		
CALR	Calreticulin	Calcium-binding chaperone that promotes folding, oligomeric assembly, and quality control in the endoplasmic reticulum (ER).
CLIC4	Chloride intracellular channel protein 4	Can insert into membranes and form poorly selective ion channels that may also transport chloride ions.
FASN	Fatty acid synthase	Catalyzes the formation of long-chain fatty acids from acetyl-CoA, malonyl-CoA, and NADPH.
MT1G	Metallothionein-1G	Negative regulation of growth
*Glucose catabolic process*		
ENO1	Alpha-enolase	The multifunctional enzyme, as well as its role in glycolysis, plays a part in various processes such as growth control, hypoxia tolerance, and allergic responses.
G6PD	Glucose-6-phosphate 1-dehydrogenase	Catalyzes the rate-limiting step of the oxidative pentose-phosphate pathway.
GPI	Glucose-6-phosphate isomerase	Glycolytic enzyme.
LDHA	L-lactate dehydrogenase A chain	Involved in step 1 of the subpathway that synthesizes (S)-lactate from pyruvate
PGD	6-phosphogluconate dehydrogenase, decarboxylating	Catalyzes the oxidative decarboxylation of 6-phosphogluconate to ribulose 5-phosphate and CO2, with concomitant reduction of NADP to NADPH
PHGDH	D-3-phosphoglycerate dehydrogenase	Catalyzes the reversible oxidation of 3-phospho-D-glycerate to 3-phosphonooxypyruvate, the first step of the phosphorylated L-serine biosynthesis pathway.
PKM	Pyruvate kinase PKM	The glycolytic enzyme that catalyzes the transfer of a phosphoryl group from phosphoenolpyruvate (PEP) to ADP, generating ATP.
TPI1	Triosephosphate isomerase	Involved in the pathway gluconeogenesis, which is part of Carbohydrate biosynthesis
PRDX5	Peroxiredoxin-5, mitochondrial	NADP metabolic process
*Protein stabilization*		
CALR	Calreticulin	Calcium-binding chaperone that promotes folding, oligomeric assembly, and quality control in the endoplasmic reticulum (ER).
HSPD1	60 kDa heat shock protein, mitochondrial	Chaperonin is implicated in mitochondrial protein import and macromolecular assembly.
LAMP2	Lysosome-associated membrane glycoprotein 2	Plays an important role in chaperone-mediated autophagy
PPIB	Peptidyl-prolyl cis-trans isomerase B	Accelerates the folding of proteins during synthesis.
ERO1A	ERO1-like protein alpha	Oxidoreductase is involved in disulfide bond formation in the endoplasmic reticulum.
*Microtubule-based transport*		
COPG1	Coatomer subunit gamma-1	Mediates biosynthetic protein transport from the ER, via the Golgi up to the trans-Golgi network.
LRPPRC	Leucine-rich PPR motif-containing protein, mitochondrial	May play a role in RNA metabolism in both nuclei and mitochondria.
RAB1A	Ras-related protein Rab-1A	The small GTPases Rab are key regulators of intracellular membrane trafficking, from the formation of transport vesicles to their fusion with membranes.
UCHL1	Ubiquitin C-Terminal Hydrolase L1	This enzyme is a thiol protease that hydrolyzes a peptide bond at the C-terminal glycine of ubiquitin.
*Protein export from the nucleus*		
CALR	Calreticulin	Calcium-binding chaperone that promotes folding, oligomeric assembly, and quality control in the endoplasmic reticulum (ER).
CSE1L	Exportin-2	Export receptor for importin-alpha.
EIF5A	Eukaryotic translation initiation factor 5A-1	mRNA-binding protein involved in translation elongation.
*Regulation of acute inflammatory response*		
ANXA1	Annexin A1	Plays important roles in the innate immune response as an effector of glucocorticoid-mediated responses and regulator of the inflammatory process.
C1QBP	Complement component 1 Q subcomponent-binding protein, mitochondrial	Involved in inflammation and infection processes, ribosome biogenesis, regulation of apoptosis, transcriptional regulation, and pre-mRNA splicing
CD59	CD59 glycoprotein	Potent inhibitor of the complement membrane attack complex (MAC) action.
PCBP2	Poly(rC)-binding protein 2	Single-stranded nucleic acid-binding protein that binds preferentially to oligo dC.

**Table 2:** Proteins which expression was altered between HO and H3.

**A: UPREGULATED**		
*Acronym*	*Name*	*Function*
*Cytoskeleton*		
CALD1	Caldesmon 1	Calmodulin- and actin-binding protein that plays an essential role in the regulation of actin/myosin interactions.
*Transcription/Translation Regulation*		
ILF2	Interleukin enhancer-binding factor 2	Transcription factor, also involved in RNA transfer from the nucleus to the cytoplasm
RPS2	40S ribosomal protein S2	Ribosome component, small subunit
RPS6	40S ribosomal protein S6	Ribosome component, small subunit, involved in the selective translation of particular classes of mRNA
RPS20	40S ribosomal protein S20	Ribosome component, small subunit
RPL18A	60S ribosomal protein L18a	Ribosome component, large subunit
RPL22	60S ribosomal protein L22	Ribosome component, large subunit
SEC61A1	Protein transport protein Sec61 subunit alpha isoform 1	Plays a crucial role in the insertion of secretory and membrane polypeptides into the ER.
*Ionic balance regulation*		
ANXA6	Annexin A6	Regulate the release of Ca2+ from intracellular stores.
*Interaction between the cell and its environment*		
CD151	CD151 antigen	Cell surface protein, involved in adhesion, mobility, integrin trafficking
**B: DOWNREGULATED**		
*Acronym*	*Name*	*Function*
*Energy metabolism*		
SLC16A3	Monocarboxylate transporter 4	Monocarboxylate transporter 4
GBE1	1,4-alpha-glucan-branching enzyme	1,4-alpha-glucan-branching enzyme
*Nucleotide biosynthesis*		
PAICS	Multifunctional protein ADE2	Multifunctional protein ADE2
CTPS1	CTP synthase 1	CTP synthase 1
*Interaction between the cell and its environment*		
EMP3	Epithelial membrane protein 3	Epithelial membrane protein 3
*RedOx regulation*		
GSTP1	Glutathione S-transferase P	Glutathione S-transferase P

**Table 3:** Proteins which expression was altered between H3 and H6.

**A: UPREGULATED**		
*Acronym*	*Name*	*Function*
*Cytoskeleton*		
S100A6	Protein S100-A6	Calcium sensor and modulator. Indirectly play a role in the reorganization of the actin cytoskeleton and in cell motility.
*Transcription/Translation Regulation*		
RPS11	40S ribosomal protein S11	Ribosome component, small subunit
RPL7	60S ribosomal protein L7	Ribosome component, large subunit
TGM2	Protein-glutamine gamma-glutamyltransferase 2	Catalyzes the cross-linking of proteins and the conjugation of polyamines to proteins.
*Ionic balance regulation*		
S100A6	Protein S100-A6	Calcium sensor and modulator. Indirectly play a role in the reorganization of the actin cytoskeleton and in cell motility.
*Energy Metabolism*		
TKT	Transketolase	NADP metabolic process.
TBC1D4	TBC1 domain family member 4	Can promote glucose transporter SLC2A4/GLUT4 translocation at the plasma membrane, thus increasing glucose uptake
**B: DOWNREGULATED**		
*Acronym*	*Name*	*Function*
*Cytoskeleton*		
PLEC	Plectin	Interlinks intermediate filaments with microtubules and microfilaments. Anchors intermediate filaments to desmosomes or hemidesmosomes.
*Transcription/Translation Regulation*		
RPS4X	40S ribosomal protein S4, X isoform	Ribosome component, large subunit
RPL22	60S ribosomal protein L22	Ribosome component, large subunit
PDIA4	Protein disulfide-isomerase A4	Catalyzes the rearrangement of -S-S- bonds in proteins.
*Interaction between the cell and its environment*		
CD151	CD151 antigen	Essential for the proper assembly of the glomerular and tubular basement membranes in the kidney.

**Table 4:** Proteins which expression was altered between H6 and H12.

**A: UPREGULATED**		
*Acronym*	*Name*	*Function*
*Cytoskeleton*		
MSN	Moesin	Member of the ERM family, functions as cross-linkers between plasma membranes and actin-based cytoskeletons
ZYX	Zyxin	Mediates adhesion-stimulated changes in gene expression and modulates the organization of actin bundles.
*Transcription/Translation Regulation*		
RPS4X	40S ribosomal protein S4, X isoform	Ribosome component, small subunit
RPS15	40S ribosomal protein S15	Ribosome component, small subunit
RTN4	Reticulon-4	Regulates membrane morphogenesis in the ER, nuclear pore complex formation, and proper localization of inner nuclear membrane proteins
EEF1G	Elongation factor 1-gamma	Probably plays a role in anchoring the complex to other cellular components, translational elongation
*Interaction between the cell and its environment*		
CLTC	Clathrin heavy chain 1	Clathrin is the major protein of the polyhedral coat of coated pits and vesicles.
ZYX	Zyxin	Mediates adhesion-stimulated changes in gene expression and modulates the organization of actin bundles.
**B: DOWNREGULATED**		
*Acronym*	*Name*	*Function*
*Intracellular Transport*		
SLC7A5	Large neutral amino acids transporter small subunit 1	High-affinity transport of large neutral amino acids.
TMED2	Transmembrane emp24 domain-containing protein 2	Involved in vesicular protein trafficking.
*Interaction between the cell and its environment*		
FN1	Fibronectin	Involved in cell adhesion, cell motility, and maintenance of cell shape.

**Table 5:** Proteins which expression was altered between H12 and H19.

**A: UPREGULATED**		
*Acronym*	*Name*	*Function*
*Energy metabolism*		
PSAP	Prosaposin	Localize primarily to the lysosomal compartment where they facilitate the catabolism of glycosphingolipids with short oligosaccharide groups.
PRKCSH	Glucosidase 2 subunit beta	Regulatory subunit of glucosidase II
*Transcription/Translation Regulation*		
ILF2	Interleukin enhancer-binding factor 2	Transcription factor, also involved in RNA transfer from the nucleus to the cytoplasm
*Interaction between the cell and its environment*		
ITGB1	Integrin beta-1	Receptors for collagen.
*Intracellular Transport*		
SLC7A5	Large neutral amino acids transporter small subunit 1	High-affinity transport of large neutral amino acids.
**B: DOWNREGULATED**		
*Acronym*	*Name*	*Function*
*Energy metabolism*		
TKT	Transketolase	NADP metabolic process.
PYGB	Glycogen phosphorylase, brain form	Glycogen phosphorylase that regulates glycogen mobilization
*Interaction between the cell and its environment*		
ANXA2	Annexin A2	Regulates membrane budding, membrane raft assembly
ZYX	Zyxin	Adhesion plaque protein.
FERMT2	Fermitin family homolog 2	Scaffolding protein that enhances integrin activation mediated by TLN1 and/or TLN2, but activates integrins only weakly by itself.
*Cytoskeleton*		
TAGLN2	Transgelin 2	Actin filament binding, the possible regulatory role
MSN	Moesin	Member of the ERM family, functions as cross-linkers between plasma membranes and actin-based cytoskeletons
S100A11	S100 Calcium Binding Protein A11	This protein may function in motility, invasion, and tubulin polymerization.
CAP1	Adenylyl cyclase-associated protein 1	Directly regulates filament dynamics, implicated in mRNA localization and the establishment of cell polarity.
IQGAP1	Ras GTPase-activating-like protein IQGAP1	Serves as an assembly scaffold for the organization of the actin cytoskeleton at the plasma membrane.
FSCN1	Fascin Actin-Bundling Protein 1	Fascin proteins organize F-actin into parallel bundles and are required for the formation of actin-based cellular protrusions.
*Ionic balance regulation*		
AHNAK	AHNAK Nucleoprotein	May be required for neuronal cell differentiation. regulation of RNA splicing
*Transcription/Translation Regulation*		
EEF1A1P5	Eukaryotic Translation Elongation Factor 1 Alpha 1 Pseudogene 5	Translation elongation factor
EEF1G	Elongation factor 1-gamma	Translation elongation factor
RPS13	40S ribosomal protein S13	Ribosome component, small subunit
RPS15	40S ribosomal protein S15	Ribosome component, small subunit
RPS24	40S ribosomal protein S24	Ribosome component, small subunit
RPL18	60S ribosomal protein L18	Ribosome component, large subunit
RPL13	60S ribosomal protein L13	Ribosome component, large subunit
SARS	Serine--tRNA ligase, cytoplasmic	Catalyzes the attachment of serine to tRNA(Ser).
PABPC4	Polyadenylate-binding protein 4	May be involved in cytoplasmic regulatory processes of mRNA metabolism.
*Intracellular Transport*		
ARF4	ADP Ribosylation Factor 4	Plays a role in vesicular trafficking and as an activator of phospholipase D
DYNC1H1	Cytoplasmic dynein 1 heavy chain 1	Acts as a motor for the intracellular retrograde motility of vesicles and organelles along microtubules.
*RedOx regulation*		
PRDX6	Peroxiredoxin-6	Involved in redox regulation of the cell

**Table 6:** Proteins which expression was altered between H19 and H24.

**A: UPREGULATED**		
*Acronym*	*Name*	*Function*
*Cytoskeleton*		
S100A6	Protein S100-A6	Calcium sensor and modulator. Indirectly play a role in the reorganization of the actin cytoskeleton and in cell motility.
*Transcription/Translation Regulation*		
H2AFX	H2A Histone Family Member X	Variant histone H2A that replaces conventional H2A in a subset of nucleosomes.
*Ionic balance regulation*		
S100A6	Protein S100-A6	Calcium sensor and modulator. Indirectly play a role in the reorganization of the actin cytoskeleton and in cell motility.
**B: DOWNREGULATED**		
*Acronym*	*Name*	*Function*
*Energy metabolism*		
TBC1D4	TBC1 domain family member 4	Can promote glucose transporter SLC2A4/GLUT4 translocation at the plasma membrane, thus increasing glucose uptake
*Cytoskeleton*		
SPTBN1	Spectrin beta chain, non-erythrocytic 1	Spectrin is an actin crosslinking and molecular scaffold protein that links the plasma membrane to the actin cytoskeleton.
PDLIM7	PDZ and LIM domain protein 7	May function as a scaffold on which the coordinated assembly of proteins can occur.
SNU13	Spectrin alpha chain, non-erythrocytic 1	Spectrin is an actin crosslinking and molecular scaffold protein that links the plasma membrane to the actin cytoskeleton.
*Transcription/Translation Regulation*		
EEF1G	Elongation factor 1-gamma	Translation elongation factor
RPS2	40S ribosomal protein S2	Ribosome component, small subunit
RPS11	40S ribosomal protein S11	Ribosome component, small subunit
RPL7	60S ribosomal protein L7	Ribosome component, large subunit
ILF2	Interleukin enhancer-binding factor 2	Transcription factor, also involved in RNA transfer from the nucleus to the cytoplasm
*Proteasome*		
PSMD3	26S proteasome non-ATPase regulatory subunit 3	Component of the 26S proteasome, a multiprotein complex involved in the ATP-dependent degradation of ubiquitinated proteins.

**Table 7:** Proteins which expression was altered between H24 and R6.

**A: UPREGULATED**		
None.		
**B: DOWNREGULATED**		
*Acronym*	*Name*	*Function*
*Energy metabolism*		
GAPDH	Glyceraldehyde-3-phosphate dehydrogenase	Catalyzes the reversible oxidative phosphorylation of glyceraldehyde-3-phosphate in the presence of inorganic phosphate and nicotinamide adenine dinucleotide.
ALDOA	Fructose-bisphosphate aldolase A	Plays a key role in glycolysis and gluconeogenesis.
*Interaction between the cell and its environment*		
FN1	Fibronectin	Involved in cell adhesion, cell motility, and maintenance of cell shape.
CLTC	Clathrin heavy chain 1	Clathrin is the major protein of the polyhedral coat of coated pits and vesicles.
*Cytoskeleton*		
MYH9	Myosin-9	Plays a role in cytokinesis, cell shape, and specialized functions such as secretion and capping. During cell spreading, plays an important role in cytoskeleton reorganization, focal contacts formation, and lamellipodial retraction; this function is mechanically antagonized by MYH10
FLNA	Filamin-A	Promotes orthogonal branching of actin filaments and links actin filaments to membrane glycoproteins
CFL1	Cofilin-1	Binds to F-actin and exhibits pH-sensitive F-actin depolymerizing activity. Regulates actin cytoskeleton dynamics.
SPTAN1	Spectrin beta chain, non-erythrocytic 1	Spectrin is an actin crosslinking and molecular scaffold protein that links the plasma membrane to the actin cytoskeleton.
SPTBN1	Spectrin beta chain, non-erythrocytic 1	Spectrin is an actin crosslinking and molecular scaffold protein that links the plasma membrane to the actin cytoskeleton.
*Ionic balance regulation*		
AHNAK	AHNAK Nucleoprotein	May be required for neuronal cell differentiation. regulation of RNA splicing
*Transcription/Translation Regulation*		
EEF1A1P5	Eukaryotic Translation Elongation Factor 1 Alpha 1 Pseudogene 5	Translation elongation factor
HNRNPA2B1	Heterogeneous nuclear ribonucleoproteins A2/B1	Heterogeneous nuclear ribonucleoprotein (hnRNP) that associates with nascent pre-mRNAs, packaging them into hnRNP particles
HNRNPD	Heterogeneous nuclear ribonucleoprotein D0	Heterogeneous nuclear ribonucleoprotein (hnRNP) that associates with nascent pre-mRNAs, packaging them into hnRNP particles
IPO5	Importin-5	Functions in nuclear protein import as nuclear transport receptor.
PTRF	Caveolae Associated Protein 1	Enables the dissociation of paused ternary polymerase I transcription complexes from the 3’ end of pre-rRNA transcripts.
PTBP1	Polypyrimidine tract-binding protein 1	Plays a role in pre-mRNA splicing and in the regulation of alternative splicing events.
RPS2	40S ribosomal protein S2	Ribosome component, small subunit
RPS4X	40S ribosomal protein S4, X isoform	Ribosome component, small subunit
RPS5	40S ribosomal protein S5	Ribosome component, small subunit
RPS8	40S ribosomal protein S8	Ribosome component, small subunit
RPS13	40S ribosomal protein S13	Ribosome component, small subunit
RPL3	60S ribosomal protein L3	Ribosome component, large subunit
RPL4	60S ribosomal protein L4	Ribosome component, large subunit
RPL6	60S ribosomal protein L6	Ribosome component, large subunit
RPL7	60S ribosomal protein L7	Ribosome component, large subunit
RPL13	60S ribosomal protein L13	Ribosome component, large subunit
RPL18	60S ribosomal protein L18	Ribosome component, large subunit, involved in translational initiation
RPL18A	60S ribosomal protein L18a	Ribosome component, large subunit
RPL23	60S ribosomal protein L23	Ribosome component, large subunit, possesses ubiquitin ligase inhibitor activity
RPL28	60S ribosomal protein L28	Ribosome component, large subunit, positive regulation of protein targeting to the mitochondrion, regulation of mitophagy
SSB	Ribosome-associated molecular chaperone SSB1	Ribosome-bound, Hsp70-type chaperone that assists in the cotranslational folding of newly synthesized proteins in the cytosol.
HSPB1	Heat shock protein beta-1	Functions as a molecular chaperone probably maintaining denatured proteins in a folding-competent state
H2AFX	H2A Histone Family Member X	Variant histone H2A replaces conventional H2A in a subset of nucleosomes.
ILF2	Interleukin enhancer-binding factor 2	may regulate transcription of the IL2 gene during T-cell activation. It can also promote the formation of stable DNA-dependent protein kinase holoenzyme complexes on DNA.
PABPC4	Polyadenylate-binding protein 4	May be involved in cytoplasmic regulatory processes of mRNA metabolism.
SND1	Staphylococcal nuclease domain-containing protein 1	Functions as a bridging factor between STAT6 and the basal transcription factor.

## Data Availability

The data presented in this study are available on request from the corresponding author. The data are not publicly available due to the nature of the nature of the experiments and the format of the public repositories.

## Supplementary Materials

The following are available online at https://www.mdpi.com/1422-0067/22/5/2384/s1, Figure S1: Number of proteins considered.

## Abbreviations

CIT	cold ischemia time
DCD	deceased after circulatory death donors
ECD	extended criteria donors
HRGEC	human renal glomerular endothelial cells
IRI	ischemia reperfusion injury
