# Supplementary figures and images for: High Throughput Proteomic Exploration of Hypothermic Preservation Reveals Active Processes within the Cell Associated with Cold Ischemia Kinetic
Source: Int J Mol Sci. 2021 Feb 27;22(5):2384. doi: 10.3390/ijms22052384 (PMC7956856; doi:10.3390/ijms22052384)

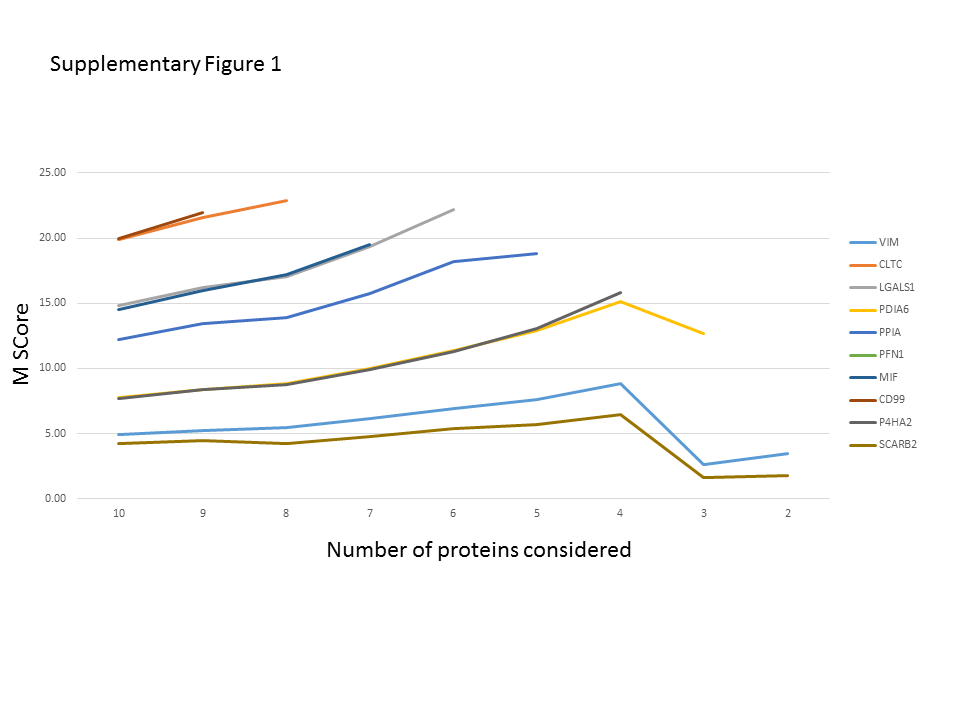

Supplement: Supplementary file 1 [file ijms-22-02384-s001.tif]
